# Critical Cooling Rate of Fast-Crystallizing Polyesters: The Example of Poly(alkylene *trans*-1,4-cyclohexanedicarboxylate)

**DOI:** 10.3390/polym16192792

**Published:** 2024-10-01

**Authors:** Kylian Hallavant, Michelina Soccio, Giulia Guidotti, Nadia Lotti, Antonella Esposito, Allisson Saiter-Fourcin

**Affiliations:** 1 INSA Rouen Normandie, CNRS, Groupe de Physique des Matériaux UMR 6634, University Rouen Normandie, F-76000 Rouen, France; kylian.hallavant@univ-rouen.fr (K.H.); allison.saiter@univ-rouen.fr (A.S.-F.); 2 Department of Civil, Chemical, Environmental and Materials Engineering, University of Bologna, Via Terracini 28, 40131 Bologna, Italy; m.soccio@unibo.it (M.S.); giulia.guidotti9@unibo.it (G.G.); nadia.lotti@unibo.it (N.L.); 3 Interdepartmental Center for Industrial Research on Advanced Applications in Mechanical Engineering and Materials Technology, CIRI-MAM, Viale del Risorgimento 2, 40136 Bologna, Italy; 4 Interdepartmental Center for Industrial Research on Buildings and Construction, CIRI-EC, Via del Lazzaretto 15/5, 40131 Bologna, Italy; 5 Interdepartmental Center for Agro-Food Research, CIRI-AGRO, Via Quinto Bucci 336, 47521 Cesena, Italy

**Keywords:** crystallization kinetics, odd-even effect, microstructure control, quenching

## Abstract

Controlling the cooling rate experienced by a material during a manufacturing process is a challenge and a major issue. Industrial processing techniques are very diverse and may involve a whole range of cooling rates, which are sometimes extremely high for small and/or thin manufactured parts. For polymers, the cooling rate has consequences on both the microstructure and the time-dependent properties. The common cooling rates associated with conventional calorimetric measurements are generally limited to a few tens of degrees per minute. This work combines several calorimetric techniques (DSC, modulated-temperature DSC, stochastically-modulated DSC and Fast Scanning Calorimetry) to estimate the critical cooling rate required to melt-quench fast-crystallizing polyesters to their fully amorphous state, based on the example of a series of poly(alkylene *trans*-1,4-cyclohexanedicarboxylate) (PCHs) with a number of methylene groups in the main structure of the repeating unit $n_{CH_{2}}$ varying from 3 to 6. The even-numbered ones require faster cooling rates (about 3000 K s^−1^ for $n_{CH_{2}}$ = 4, between 500 and 1000 K s^−1^ for $n_{CH_{2}}$ = 6) compared to the odd-numbered ones (between 50 K min^−1^ and 100 K s^−1^ for $n_{CH_{2}}$ = 3, between 10 and 30 K min^−1^ for $n_{CH_{2}}$ = 5).

## 1. Introduction

Polymers play an increasing role in manufacturing processes and their production keeps on growing, especially in the packaging industry [1], which is the biggest consumer and producer of plastic waste. However, the decline of fossil resources, and the raise of collective awareness about the impact that waste has on the environment, impose to look for possible alternatives to petroleum-based polymers with reduced carbon footprint and environmental risks. Plastic waste started accumulating in increasingly large amounts in the fifties, and nowadays the environmental pollution due to plastic waste is becoming severe. For this reason, bio-sourced and/or biodegradable polymers, in particular polyesters, have attracted much attention from both academic researchers and industrials—because they appear as one of the most promising solutions for next-generation sustainable packaging, with improved barrier properties against gas and water vapor, and balanced mechanical properties [2,3].

**Figure 1:**
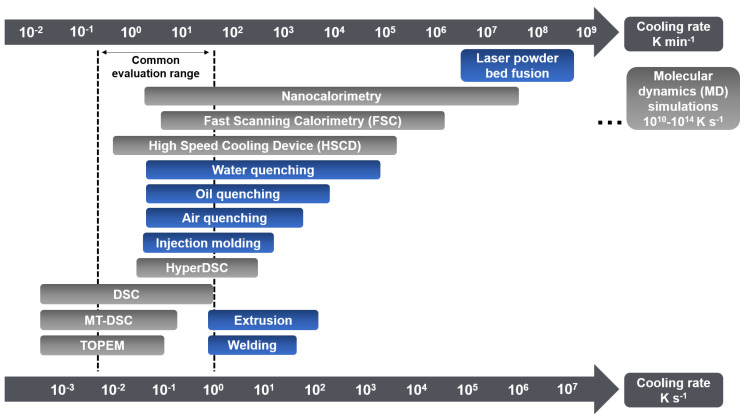
Typical range of cooling rates achievable with different processing and characterization techniques for different materials including polymers [5,6,7,8,9,10,11,12,13,14,15,16,17,18,19,20,21,22,23,24].

Industrial processing techniques, such as injection molding or additive manufacturing (laser bed fusion, 3D-printing…), involve a whole range of cooling rates (Figure 1), which depends on the surface-to-volume ratio of the manufactured item, the cooling technology and the temperature of the cooling medium, the covered temperature range, and the thermal conductivity of the material. Sometimes the cooling rates experienced by the material are extremely high, especially when the manufactured parts are small and/or thin. Of course, increasing the cooling rates used for processing helps increasing cost-effectiveness, however it is appropriate to recall that cooling may also have major consequences on polymer microstructure and molecular mobility [4]. In any case, controlling the local cooling rate and understanding its consequences on polymer properties remain a major challenge.

A series of biodegradable and potentially bio-based thermoplastic polyesters has been recently synthesized from *trans*-1,4-cyclohexanedicarboxylic acid and linear diols of different lengths. These materials showed interesting barrier and mechanical properties for food packaging applications [25,26,27,28,29], but also a very different aptitude to crystallize depending on the number of methylene groups -CH_2_- in their repeating unit (odd-even effect) [30,31,32,33,34,35]. The cooling rate required for full quenching is expected to be very different for each one of these materials, with different consequences for the ones that are able to crystallize compared to the ones that cannot [36]. It has already been shown that, besides making a difference in terms of nucleus density [37], crystal fraction [38], crystallite size, lamellar thickness and spherulite morphology [39], playing on the cooling rate may significantly affect physical ageing [40] and consequently any other time-dependent property [41,42], even when the polymers are melt-quenched to their fully amorphous state and kept at temperatures below their glass transition.

Schawe and Löffler [43] took the example of Au-based glasses to discuss the existence of multiple critical cooling rates for the generation of different types of glasses. They identified three thresholds, which they called $\beta_{c,SCG}$ (critical cooling rate to obtain a semi-crystalline glass), $\beta_{c,SDG}$ (critical cooling rate to obtain a self-doped glass), and $\beta_{c,CHG}$ (critical cooling rate to obtain a chemically-homogeneous glass). Transposing and adapting this terminology to macromolecular glasses, one may expect that (1) increasing the cooling rate is an efficient way to melt-quench polymers that are intrinsically able to crystallize, (2) crystallization is observed only if the cooling rate is kept below a critical value $\beta_{c,SDG}$, (3) crystallinity reaches different extents depending on the applied cooling rate $\beta_{c,SCG}$ (faster cooling reduces it since it shortens the time allowed for chain folding and vice-versa), (4) because of chain entanglements, crystallinity increases as the cooling rate decreases, until hitting a plateau (100% crystallinity is never reached, not even at the lowest values of $\beta_{c,SCG}$ or in isothermal conditions), (5) when the cooling rate reaches the threshold $\beta_{c,SDG}$, a self-doped glass (SDG) is obtained, and (6) it is necessary to reach values of cooling rate at least equal to a further threshold $\beta_{c,CHG}$ to obtain a so-called chemically-homogeneous glass (CHG). Cooling down crystallizable polymers at different cooling rates $\beta_{c}<\beta_{c,SDG}$ may affect their crystalline phase not only quantitatively, but also qualitatively, worsening the intrinsic complexity due to chemistry (polymorphism [44,45,46,47], imperfect chain folding with extended coupling between the crystalline and amorphous domains [48,49,50], development of mesophases [51,52]…). In general, reducing the cooling rate favors the development of larger amounts of more perfect crystals, with positive consequences on properties such as the Young’s modulus [53], whereas increasing the cooling rate improves transparency, toughness and elongation at break [54].

Conventional calorimetric measurements are performed at scanning rates generally limited to a few tens of degrees per minute. Fast Scanning Calorimetry (FSC) allows the extension of the experimental window of several orders of magnitude in terms of scanning rate, giving access to cooling rates up to 40,000 K s^−1^ [41,55], which is ideal to reproduce industrial processing conditions (Figure 1). This work illustrates how to estimate the critical cooling rate $\beta_{c,SDG}$ (threshold between semi-crystalline and fully amorphous samples obtained during melt-quenching) for fast-crystallizing polyesters, taking the example of a series of poly(alkylene *trans*-1,4-cyclohexanedicarboxylate) (PCHs) with a number of methylene groups in the main structure of the repeating unit $n_{CH_{2}}$ varying from 3 to 6, and combining several calorimetric techniques, i.e., Differential Scanning Calorimetry (DSC), stochastically-modulated DSC (TOPEM) and Fast Scanning Calorimetry (FSC).

## 2. Materials and Methods

### 2.1. Reagents

*Trans*-1,4-cyclohexanedicarboxylic acid (95%, 5 mol% *cis*-isomers) (CHDA) was purchased from Fluorochem (Hadfield, UK). 1,3-propanediol (PD) (98%) was purchased from Carbosynth. 1,4-butanediol (BD) (99%) was purchased from Sigma-Aldrich. 1,5-pentanediol (PeD) (97%) was purchased from Fluka Chemika. 1,6-hexanediol (HD) (>97%) was purchased from TCI. Titanium tetrabutoxide Ti(OBu)_4_ (TBT) was purchased from Sigma-Aldrich. All reagents were used as received.

### 2.2. Polymer Synthesis

Four poly(alkylene *trans*-1,4-cyclohexanecarboxylate)s (PCHs) were synthesized by a two-step solvent-free melt polycondensation procedure consisting in the esterification reaction between a bifunctional alicyclic acid (CHDA) and a bifunctional linear aliphatic glycol. Depending on the selected glycol, four homopolymers were obtained with a variable number of methylene groups $n_{CH_{2}}$ in the glycolic fraction of their repeating unit. Poly(propylene *trans*-1,4-cyclohexanedicarboxylate) (PPCE) with $n_{CH_{2}}$ = 3 was obtained with PD. Poly (butylene *trans*-1,4-cyclohexane dicarboxylate) (PBCE) with $n_{CH_{2}}$ = 4 was obtained with BD. Poly(pentamethylene *trans*-1,4-cyclohexanedicarboxylate) (PPeCE) with $n_{CH_{2}}$ = 5 was obtained with PeD. Poly (hexamethylene *trans*-1,4-cyclohexanedicarboxylate) (PHCE) with $n_{CH_{2}}$ = 6 was obtained with HD. Each synthesis was carried out starting from CHDA, the selected diol (100% molar excess with respect to the diacid), and TBT (200 ppm) as catalyst, within a 250-mL glass reactor stirred at about 50 rpm in a thermostated oil bath connected to a six-bulb condenser to prevent the evaporation of the reagents. The molar excess of diol promoted the dissolution of the diacid. In the first step, the temperature was set to 190 °C and kept constant for about 1.5 h under a continuous flow of pure nitrogen (50 mL min^−1^) and a pressure of 1 atm, allowing to distill off up to 90% of the theoretical amount of water. At the beginning of the second step, stirring was increased to 100 rpm and the temperature was raised to 200 °C to remove the excess of diol, then the temperature was further raised to 210 °C and the pressure was gradually reduced to 0.06 mbar to promote the transesterification reactions. The synthesis was carried out for 2 h, until a high and constant torque value was measured indicating that a high molecular weight was reached, and no more distillation was observed. After the synthesis, all the PCHs were purified by dissolution in chloroform and precipitation in a beaker filled with methanol in large excess (chloroform:methanol 1:10). After purification by dissolution/precipitation, the samples were dried under vacuum (1 h at 0.1 mbar). The samples were continuously stored under vacuum at room temperature, in a dessicator with phosphorus pentoxide P_2_O_5_ to reduce exposure to humidity until characterization.

### 2.3. Chemical Characterizations

H1-NMR analyses were carried out to confirm the chemical structure and the *cis*/*trans* isomer ratio of the synthesized PCHs. The spectra were acquired using a Varian XL-400 NMR spectrometer (Palo Alto, CA, USA) at room temperature (relaxation time = 0 s, acquisition time = 1 s, 100 repetitions). The polymers were dissolved by introducing about 15 mg of sample in 1 mL of deuterated chloroform (containing 0.03 % tetramethylsilane, TMS, as internal reference). The number-average molecular weight (${\bar{M}}_{n}$), the weight-average molecular weight (${\bar{M}}_{w}$), the dispersity (*Đ*) and the degree of polymerization (DP_*n*_) of the samples were estimated thanks to the data obtained by Gel Permeation Chromatography (GPC) analysis performed at 30 °C using an HPLC 1100 chromatograph (Agilent Technologies, Santa Clara, CA, USA) equipped with a PLgel 5 mm MiniMIX-C column. A chloroform solution was used as eluent with a flow of 0.3 mL min^−1^, and sample concentrations of about 2 mg mL^−1^ were adopted. The calibration curve was obtained using polystyrene standards with a molecular range of 800–100,000 g mol^−1^. H1-NMR spectra of the four polyesters can be found in the Supporting Information (Figures S1–S4). A summary of the chemical features of the investigated PCHs is given in Table 1.

### 2.4. Thermal Characterizations

Given the aim of the study, the thermal characterizations mostly consisted in calorimetric investigations. Different equipments were used to cover the largest possible range of cooling rates. The lowest cooling rates were covered by Differential Scanning Calorimetry (DSC) and stochastically-modulated DSC (TOPEM), whereas the highest cooling rates were covered by Fast Scanning Calorimetry (FSC) with either UFS1 (lower range) or UFH1 (upper range) MultiSTAR sensors.

DSC and TOPEM experiments were performed using a DSC 3+ (Mettler Toledo) equipped with a FRS 6+ sensor and piloted by the STARe software. Calibrations for temperature, enthalpy, and thermal lag (tau lag) were achieved using zinc, indium, and water standards. The samples for both DSC and TOPEM (masses comprised between 2 and 10 mg) were placed in 40 µL sealed aluminum pans. The measurements were conducted under a constant nitrogen flow (50 mL min^−1^). TOPEM measurements were performed with a pulse height of 0.1 K and pulse widths stochastically varying between 15 and 30 s, then an extrapolation to zero frequency was performed to obtain the quasi-static heat capacity $c_{p,0}$ as a function of temperature. More details about TOPEM can be found elsewhere [56].

*FSC* measurements were performed using a Flash DSC 2+ (Mettler Toledo) equipped with a Huber intracooler TC100 and also piloted by the STARe software. The samples for *FSC* (masses comprised between 10 and 200 ng) were obtained by successively cutting bulk samples into smaller pieces under a microscope, and then transferred by a hair of a paint brush onto the center of the active zone of a conditioned and temperature-corrected MultiSTAR sensor. The measurements were conducted under a constant argon flow (60 mL min^−1^). The sensor support temperature was set at −95 °C.

For samples that could be successfully melt-quenched by conventional *DSC*, the mass deposited onto the *FSC* sensor was estimated according to Equation (1), i.e., by comparing the change in heat capacity at the glass transition temperature $\Delta C_{p}$ obtained with *FSC* and *DSC*, as previously reported in the literature [40].

(1) $$m=\frac{\Delta C_{p}^{FSC}}{\Delta c_{p}^{DSC}}$$

where $\Delta C_{p}^{FSC}$ [J K^−1^] is the heat capacity step estimated by *FSC* and $\Delta c_{p}^{DSC}$ [J g^−1^ K^−1^] is the heat capacity step measured by *DSC*, both at the glass transition temperature.

For samples that could not be obtained in their fully amorphous state within the cooling range provided by conventional *DSC*, the mass deposited onto the *FSC* sensor was estimated by comparing the values of heat capacity measured by *FSC* and specific heat capacity measured by TOPEM at the same temperature, according to Equation (2).

(2) $$m=\frac{C_{p}^{FSC}\left(T\right)}{c_{p}^{TOPEM}\left(T\right)}$$

where $C_{p}^{FSC}$ [J K^−1^] is the heat capacity measured by *FSC* and $c_{p}^{TOPEM}$ [J g^−1^ K^−1^] is the specific heat capacity measured by TOPEM at the same temperature T. At least two temperatures were selected, one in the glassy and one in the liquid state, and the results were averaged.

Preliminary characterizations of the thermal behavior of each PCH were done by DSC through typical heating-cooling-heating ramps, with heating and cooling rates $\beta_{h}=\left|\beta_{c}\right|$ = 10 K min^−1^. The first heating ramp brought the samples up to the melt, thus ensuring the best thermal contact with the bottom of the aluminum pan and erasing any previous thermal history. The subsequent cooling ramp allowed to compare the relative aptitude to crystallize when the four samples were cooled from the melt at a constant arbitrary cooling rate, providing the temperature range over which crystallization is expected to occur. The second heating ramp provided a glimpse on the microstructural differences induced on each sample due to their different chemical composition, despite the common cooling conditions.

**Figure 2:**
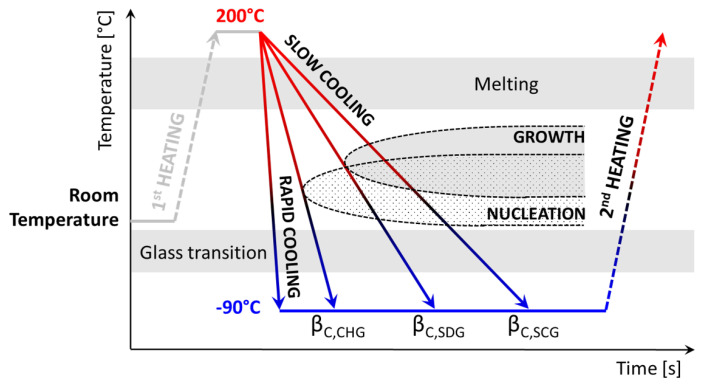
Schematic representation of the thermal protocol used to evaluate the critical cooling rate $\beta_{c,SDG}$, i.e., the minimum cooling rate allowing to obtain a fully amorphous polymer upon quenching from the molten state. The scans in terms of cooling rate covered five decades (from 2 K min^−1^ to 5000 K s^−1^) thanks to a combination of different calorimetric techniques, i.e., Differential Scanning Calorimetry (DSC), stochastically-modulated DSC (TOPEM) and Fast Scanning Calorimetry (FSC).

Based on these preliminary results, a thermal protocol consisting in a series of successive cooling and heating ramps was designed for each PCH, with the purpose of evaluating its critical cooling rate $\beta_{c,SDG}$, i.e., the minimum cooling rate at which no crystallization is observed when the polymer is cooled down from the melt. The protocol consisted in heating up each sample with a constant heating rate $\beta_{h}$ to a temperature slightly above its melting, holding it for 0.1 s to ensure that melting is complete, cooling it down through the glass transition to −90 °C at a constant cooling rate $\beta_{c}$, then heating it up again at 1000 K s^−1^ to check for any possible sign of crystals through melting. The protocol, whose temperature ranges and cooling rates were adjusted to each sample’s thermal behavior (crystallization temperature T_*c*_, glass transition temperature T_*g*_, melting temperature range $\Delta T_{m}$), is schematically represented in Figure 2. Five decades of cooling rates (from 2 K min^−1^ to 5000 K s^−1^) were investigated to assess the critical cooling rates of PPCE, PBCE, PPeCE and PHCE.

## 3. Results and Discussion

Whenever an alicyclic moiety is introduced in a polymer backbone, the *cis*/*trans* isomer ratio is expected to potentially influence its crystallization behavior. In particular, the crystallization temperature $T_{c}$, glass transition temperature $T_{g}$ and melting temperature $T_{m}$ are known to depend on the *trans*-isomer content [57]. Even though the diacid selected for this work mostly contained *trans*-isomers (with only 5% *cis*-isomers), the *cis*/*trans* isomer ratio was evaluated for all the synthesized PCHs prior to any other thermal characterization (results reported in Table 1). Indeed, it cannot be excluded that the configuration of some molecules changes from *trans* to *cis* during polymerization, the process being generally favored by long exposures to relatively high temperatures in the presence of a catalyst. To minimize the occurrence of undesired *trans*/*cis* isomerization, i.e., to maintain the highest possible content of *trans*-isomers and consequently ensure high values of $T_{c}$, the temperature, time and catalyst content used for the synthesis should be minimized. On the other hand, these very same variables also control the increase in molecular weight during polymerization. Therefore, a compromise had to be found between the best conditions to get high values of $T_{c}$, and the best conditions to get a high molecular weight [58]. The synthesis performed in this work was optimized with respect to previous batches [36], for which slightly higher *cis*-isomer contents were obtained. The highest efficiency in maintaining a fixed *trans*-isomer content was observed when CHDA was combined with BD and PeD, i.e., for the synthesis of PBCE ($n_{CH_{2}}$ = 4) and PPeCE ($n_{CH_{2}}$ = 5). The largest extent of undesired *trans*/*cis* isomerization was observed during the synthesis of PHCE ($n_{CH_{2}}$ = 6), with a *cis*-isomer content however limited to less than 10 %. Both the molecular weight and the degree of polymerization decreased as $n_{CH_{2}}$ increased, to the point that PHCE ended with a two-fold smaller molecular weight and DP_*n*_ compared to PPCE. However, all PCHs were synthesized with satisfactory molecular weights and the same dispersity *Đ* ≈ 1.5 (Table 1).

**Figure 3:**
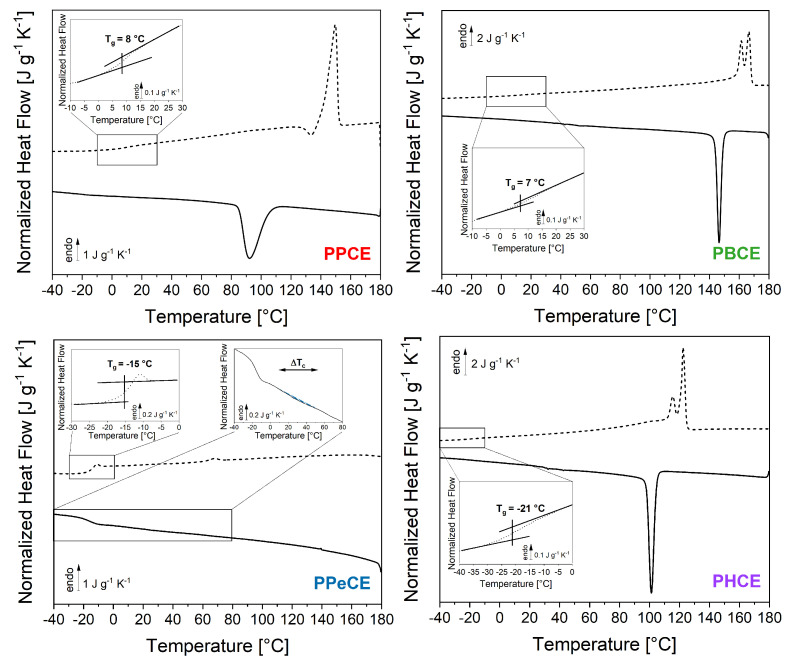
Thermal behavior recorded by DSC with cooling and heating rates $|\beta_{c}|$ = $\beta_{h}$ = 10 K min^−1^ after melting (the first heating ramp is not shown). A zoom into the glass transition temperature range is reported for each sample in the corresponding inset. For PPeCE, an additional inset zooms into the crystallization temperature range (dashed blue area). PPCE = poly(propylene *trans*-1,4-cyclohexanedicarboxylate). PBCE = poly(butylene *trans*-1,4-cyclohexanedicarboxylate) (PBCE). PPeCE = poly(pentamethylene *trans*-1,4-cyclohexanedicarboxylate). PHCE = poly(hexamethylene *trans*-1,4-cyclohexanedicarboxylate).

Figure 3 shows the preliminary results obtained by DSC on all the considered PCHs. The initial melting (first heating ramps) are not shown; the cooling ramps from the melt down to negative temperatures at $|\beta_{c}|$ = 10 K min^−1^ are represented by solid lines; the subsequent heating ramps at $\beta_{h}$ = 10 K min^−1^ are represented by dashed lines. A zoom into the glass transition temperature range is reported in the inset to each graph. The thermal characteristics evidenced by the preliminary results in Figure 3 are summarized in Table 2. The most striking result is that PBCE and PHCE, i.e., the PCHs with an even number of methylene groups ($n_{CH_{2}}$ = 4 and 6), are prone to a very rapid crystallization process ($\Delta T_{c}$ < 10 C), whereas PPCE ($n_{CH_{2}}$ = 3) crystallizes much slower and PPeCE ($n_{CH_{2}}$ = 5) barely has the time to start the process and shows a wide crystallization peak (large $\Delta T_{c}$) with a low intensity ($\Delta h_{c}$ of 0.5 J g^−1^). According to these results, the PCHs with an even value of $n_{CH_{2}}$ are expected to have the highest critical cooling rates $|\beta_{c,SDG}|$, whereas the ones with an odd value of $n_{CH_{2}}$ should be the easiest to melt-quench. In both cases, an increase in the number of methylene groups appears to profoundly change the crystallization behavior. When the number of methylene groups is even, the change is mostly seen in terms of $T_{c}$ (with a decrease of about 45 C as $n_{CH_{2}}$ increases from 4 to 6) with no significant changes in $\Delta T_{c}$. When the number of methylene groups is odd, the change is more dramatic. As $n_{CH_{2}}$ increases from 3 to 5, the temperature range for crystallization spreads from about 20 °C to more than 40 °C, and the crystal growth is almost entirely suppressed. For purposes such as minimizing the cycle time for injection molding and yet developing a semi-crystalline microstructure, PCHs with an even number of methylene groups would be the best choice, because crystallization starts at high temperatures and proceeds very fast; the subsequent heating ramp, though, suggests that the crystalline phase grown upon cooling is complex, with a marked trend to reorganization for improved crystal perfection (the melting temperature range $\Delta T_{m}$ spreads over ≈80 °C, and the melting peak has a shape that recalls the double melting peak of polyhydroxyalkanoates [59,60], which are known to be prone to extensive crystalline reorganization upon heating). Crystalline reorganization occurs also in PCHs with an odd number of methylene groups, but apparently to a less extent, with a much narrower melting temperature range ($\Delta T_{m}$ < 25 °C) and just a small exotherm preceding the melting endotherm.

Irrespective of the odd or even character of $n_{CH_{2}}$, the melting temperature generally decreases as the number of methylene groups increases. However, the lowest melting (and crystallization) temperature is observed for PPeCE ($n_{CH_{2}}$ = 5). This particular behavior has been reported for other polymers containing five methylene groups in their backbone [31,35]. This phenomenon could be due to some local polarization that builds up in odd-numbered polyesters, hindering the crystallization process, whereas in even-numbered polyesters the dipoles are aligned in opposite directions, allowing a more efficient chain folding and packing [34,61]. NMR experiments conducted on flexible alkyl chains with mesogenic groups at either end showed that, for $n_{CH_{2}}$ = 5, the alkyl chain is in an all-*trans* conformation, except that the conformation around the C-O single bonds was found to be approximately *gauche* [61]. This *gauche* conformation forces the molecule to bend more with respect to an all-*trans* conformation, causing less efficient packing and therefore improving its glass-forming ability. A recent investigation on the crystal structure of PBCE confirms the all-*trans* conformation of its alkyl segments [62]. The values of glass transition temperature $T_{g}$ consistenly decrease with the number of methylene groups introduced in the polymer backbone, from about 8 °C for PPCE ($n_{CH_{2}}$ = 3) to about −21 °C for PHCE ($n_{CH_{2}}$ = 6).

**Figure 4:**
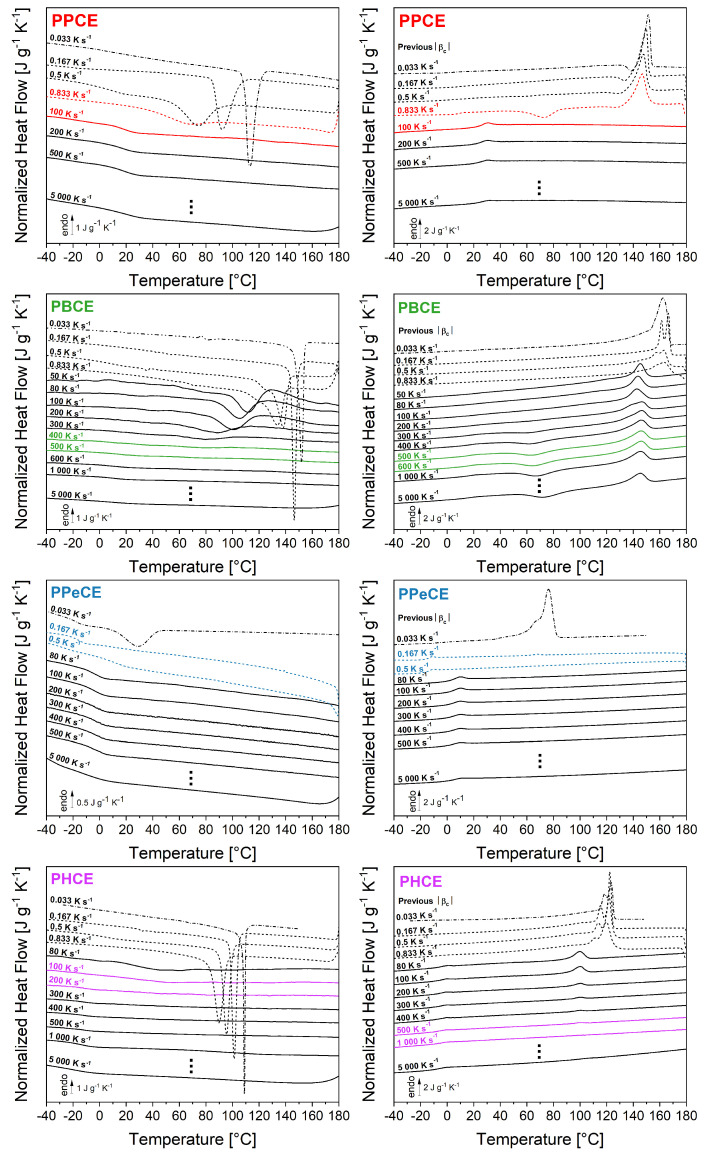
Normalized heat flow recorded upon cooling from the melt (left column) and during the subsequent heating ramp (right column). The curves at the lowest cooling rate $|\beta_{c}|$ = 0.033 K s^−1^ (dashed-dotted lines) were obtained by TOPEM. The curves plotted with dashed lines were obtained by conventional DSC. The curves at the highest cooling rates were obtained by FSC (solid lines). The heating ramps were obtained with $\beta_{h}=\left|\beta_{c}\right|$ (TOPEM and DSC) or $\beta_{h}$ = 1000 K s^−1^ (FSC). The curves in colors highlight the range within which the critical cooling rate $|\beta_{c,SDG}|$ is supposed to fall based on either crystallization (left column) or subsequent melting (right column). PPCE = poly(propylene *trans*-1,4-cyclohexanedicarboxylate). PBCE = poly(butylene *trans*-1,4-cyclohexanedicarboxylate). PPeCE = poly(pentamethylene *trans*-1,4-cyclohexanedicarboxylate). PHCE = poly(hexamethylene *trans*-1,4-cyclohexanedicarboxylate).

Figure 4 (left column) reports the cooling ramps obtained for each PCH according to the thermal protocol sketched in Figure 2. The cooling ramps recorded at the lowest cooling rate $|\beta_{c}|$ = 0.033 K s^−1^ were obtained by TOPEM (dashed-dotted lines). The cooling ramps plotted with dashed lines were obtained by conventional DSC. The cooling ramps recorded at the highest cooling rates were obtained by FSC (solid lines). A quick glance at the cooling curves confirms that PPeCE is the easiest to melt-quench, whereas PBCE is the most challenging. It is indeed necessary, at first, to check that no exothermal signals associated with crystallization are recorded during the cooling ramp. Based on this criterion, a threshold cooling rate could be identified for each PCH (left column, curves in colors). The corresponding critical cooling rates would be somewhere between 0.833 K s^−1^ (50 K min^−1^) and 100 K s^−1^ for PPCE, between 400 and 500 K s^−1^ for PBCE, between 0.167 K s^−1^ (10 K min^−1^) and 0.5 K s^−1^ (30 K min^−1^) for PPeCE, and between 100 and 200 K s^−1^ for PHCE. It is however necessary to double-check these values by considering the heating ramps recorded right after cooling.

In Figure 4 (right column), the heating ramps recorded by TOPEM and DSC were obtained with $\beta_{h}=\left|\beta_{c}\right|$, and all the heating ramps obtained by FSC were recorded at $\beta_{h}$ = 1000 K s^−1^. The double-check consists in verifying that no endothermal signals associated with melting are recorded upon heating, or that the recorded enthalpy of melting is perfectly balanced by the enthalpy of cold crystallization (if cold crystallization occurs). Based on this additional criterion, the threshold cooling rate for melt-quenching can in some cases be readjusted (right column, curves in colors). About PPCE, for instance, Figure 4 shows that (1) the slowest cooling rate leads to a fully crystallized sample (TOPEM), (2) a progressive increase in the cooling rate hinders the crystallization process and let appears cold crystallization (DSC curves), and (3) FSC allows to melt-quench the sample (no cold crystallization is observed during the subsequent heating ramp because of the higher heating rate with respect to DSC measurements). In the case of PBCE, it is worth noting that (1) only FSC is able to provide sufficiently high cooling rates for an efficient melt-quenching, (2) the range of cooling rates previously identified as critical (400–500 K s^−1^) is sufficient to suppress the crystallization from the melt and cold crystallization occurs during the subsequent heating ramps, (3) a heating rate faster than 1000 K s^−1^ is required to suppress cold crystallization and any other possible melting-recrystallization process. Focusing on the heating ramps recorded after cooling at 300, 400, 500 and 600 K s^−1^, one may notice that the peak of cold crystallization keeps on evolving, which confirms that vitrification is more and more efficient; the shape of the peak stabilizes between 500 and 600 K s^−1^, which is therefore considered as a better estimation of the range within which the critical cooling rate is supposed to fall. PPeCE is easily quenched with conventional cooling rates. As for PHCE, based solely on the cooling ramps one may guess that the critical cooling rate is between 100 and 200 K s^−1^ (left column, curves in colors), however the heating ramps clearly show that a crystalline phase is formed for cooling rates up to 500–1000 K s^−1^ (right column, curves in colors).

**Figure 5:**
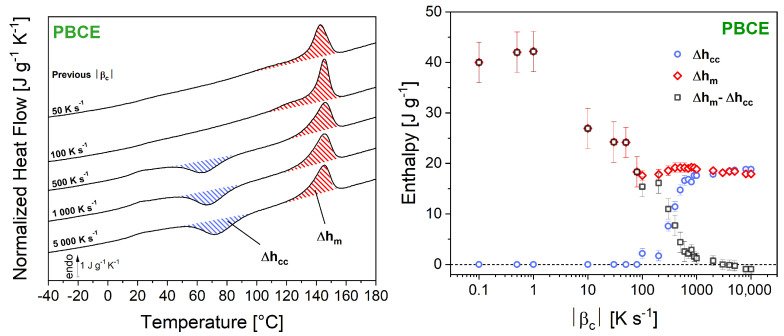
(**Left**) Calculation of the enthalpies of cold crystallization $\Delta h_{cc}$ (blue-hatched areas) and melting $\Delta h_{m}$ (red-hatched areas) for poly (butylene *trans*-1,4-cyclohexanedicarboxylate) (PBCE) previously cooled down from the melt at different cooling rates $|\beta_{c}|$. (**Right**) The enthalpies of cold crystallization $\Delta h_{cc}$ (blue circles) and melting $\Delta h_{m}$ (red diamonds), along with their algebraic difference $\Delta h_{m}-\Delta h_{cc}$ (black squares) are then plotted against the cooling rate $\beta_{c}$ previously used to attempt melt-quenching.

Of all the considered samples, PBCE is the only one being able to cold-crystallize despite the relatively high heating rates used in FSC experiments (1000 K s^−1^) (Figure 4, right column). Figure 5 illustrates the additional criterion to meet to make sure that a polymer able to cold-crystallize is completely vitrified during melt-quenching. Figure 5 (left) shows a selection of heating ramps recorded after cooling from the melt at different $|\beta_{c}|$. The additional criterion consists in calculating the enthalpy of cold crystallization $\Delta h_{cc}$ and the enthalpy of melting $\Delta h_{m}$, and then verifying if they are perfectly balanced. For cooling rates below 100 K s^−1^, no cold crystallization is observed during the subsequent heating. When faster cooling rates are used, cold crystallization occurs, and the associated enthalpy $\Delta h_{cc}$ gradually increases until reaching a plateau (18 J g^−1^ for $|\beta_{c}|$ = 1000 K s^−1^). On the other hand, the enthalpy of melting $\Delta h_{m}$ decreases from 40 to 18 J g^−1^ as the cooling rate increases up to 100 K s^−1^, and then stabilizes at 18 J g^−1^ as well. Figure 5 (right) reports the values of $\Delta h_{cc}$, $\Delta h_{m}$, and their algebraic difference $\Delta h_{m}-\Delta h_{cc}$, plotted against the cooling rate $|\beta_{c}|$ previously used for melt-quenching. With this additional criterion, the critical cooling rate $|\beta_{c,SDG}|$ for PBCE is rather estimated at about 3000 K s^−1^ (when $\Delta h_{m}-\Delta h_{cc}$ = 0).

**Figure 6:**
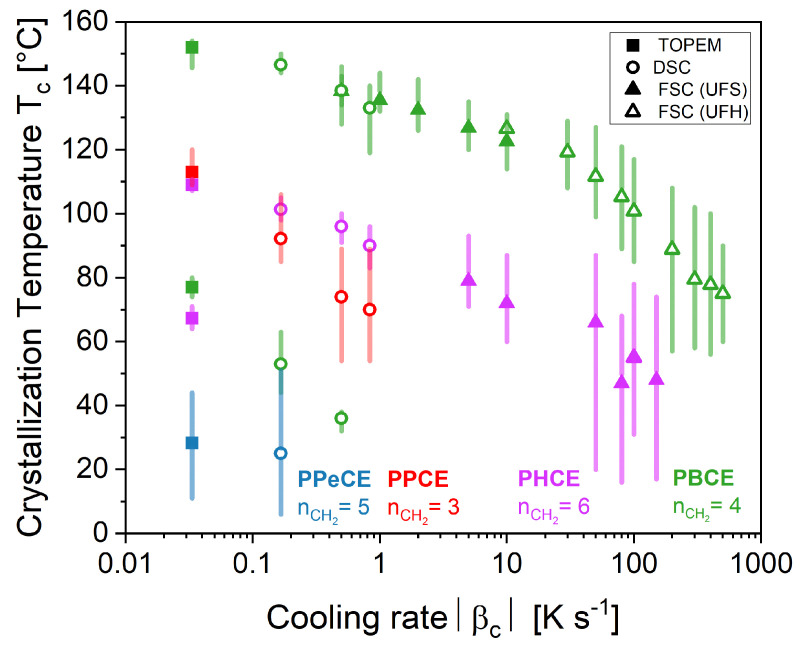
Crystallization temperatures $T_{c}$ (symbols) and the temperature ranges at which crystallization occurs $\Delta T_{cc}$ (bars) measured upon cooling from the melt at different cooling rates $|\beta_{c}|$. The bars represent the temperature range between the onset ($T_{c,on}$) and endset ($T_{c,end}$) of the crystallization peak. PPCE = poly(propylene *trans*-1,4-cyclohexanedicarboxylate). PBCE = poly(butylene *trans*-1,4-cyclohexanedicarboxylate). PPeCE = poly(pentamethylene *trans*-1,4-cyclohexanedicarboxylate). PHCE = poly(hexamethylene *trans*-1,4-cyclohexanedicarboxylate).

Figure 6 shows the influence of the cooling rate on the crystallization temperature $T_{c}$ (symbols), and more generally on the temperature range at which crystallization occurs (bars). Two phenomena are evidenced. The first observation is that, irrespective of $n_{CH_{2}}$, an increase in the cooling rate $|\beta_{c}|$ leads to a decrease in the crystallization temperature $T_{c}$ and a broadening of the crystallization peak (increase in $\Delta T_{cc}$), from less than 10 °C at the slowest cooling rates to more than 30 °C at the fastest cooling rates. The shift of $T_{c}$ and the increase in $\Delta T_{cc}$ both prove that the observed transformation is controlled by nucleation; indeed, slow cooling enables the activation of the nuclei at higher temperature, whereas fast cooling retards and slows down the nucleation process [63].

The second observation is that the odd-numbered polyesters are associated with larger crystallization peaks and lower crystallization temperatures in comparison with their even-numbered counterparts at the same cooling rate. It should also be mentioned that PBCE has recently been shown to crystallize in at least two polymorphic forms, α and β, with the α-form observed upon slow cooling, and the β-form generated with sufficiently fast cooling from the melt (however the explored range of cooling rates did not exceed 50 K min^−1^) [62]. With this in mind, the shift of $T_{c}$ from 80 to 150 °C could be interpreted as the progressive transformation of the metastable β-form into the more stable α-form as the undercooling is reduced. One may also notice that both PBCE and PHCE have a double value of $T_{c}$ at the lowest cooling rates. This could be due to either polymorphism (as previously mentioned for PBCE) or just to crystalline perfection through melting-recrystallization upon heating. So far, the literature has reported no evidence of polymorphism for PHCE.

**Figure 7:**
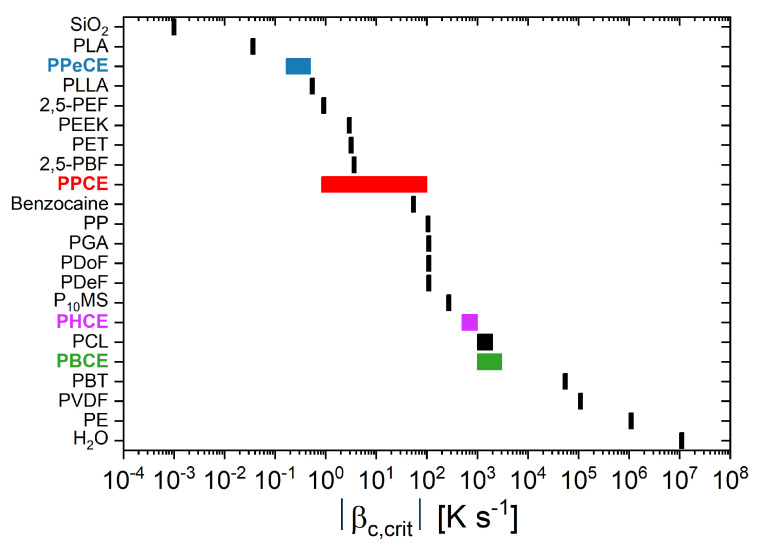
Values of critical cooling rates $|\beta_{c,crit}|$ issued from the literature [9,37,64,65,66,67,68,69,70,71] for common polymers as well as for a few other materials (silica, benzocaine, water). PPCE = poly(propylene *trans*-1,4-cyclohexanedicarboxylate). PBCE = poly(butylene *trans*-1,4-cyclohexanedicarboxylate). PPeCE = poly(pentamethylene *trans*-1,4-cyclohexanedicarboxylate). PHCE = poly(hexamethylene *trans*-1,4-cyclohexanedicarboxylate).

Figure 7 shows a summary of the critical cooling rates $|\beta_{c,crit}|$ reported in the literature for common polymers and for a few other materials (silica, benzocaine, water). The critical cooling rates estimated in this work for PPCE, PBCE, PPeCE and PHCE are also reported for comparison purposes. As expected, the even-numbered PCHs (PBCE with $n_{CH_{2}}$ = 4 and PHCE with $n_{CH_{2}}$ = 6) require faster cooling rates to be effectively melt-quenched as compared to the odd-numbered PCHs (PPCE with $n_{CH_{2}}$ = 3 and PPeCE with $n_{CH_{2}}$ = 5). It also appears that, irrespective of the odd or even character of $n_{CH_{2}}$, the value of critical cooling rate decreases as the length of the alkyl chain within the repeating unit increases, suggesting that the methylene groups act as defects for crystal formation. This observation cannot be extended to other systems, such as poly (ethylene terephthalate) (PET) and poly (butylene terephthalate) (PBT), for which an increase of $n_{CH_{2}}$ from 2 to 4 leads to a four-decade decrease in $|\beta_{c,crit}|$.

## 4. Conclusions

This work combines several calorimetric techniques, i.e., Differential Scanning Calorimetry (DSC), modulate-temperature DSC (MT-DSC), stochastically-modulated DSC (TOPEM) and Fast Scanning Calorimetry (FSC), to estimate the critical cooling rate necessary to melt-quench fast-crystallizing polyesters to their fully amorphous state. The method is applied to a series of poly(alkylene *trans*-1,4-cyclohexanedicarboxylate) (PCHs) with a number of methylene groups $n_{CH_{2}}$ varying from 3 to 6, i.e., poly(propylene *trans*-1,4-cyclohexanedicarboxylate) (PPCE), poly(butylene *trans*-1,4-cyclohexanedicarboxylate) (PBCE), poly(pentamethylene *trans*-1,4-cyclohexanedicarboxylate) (PPeCE), and poly(hexamethylene *trans*-1,4-cyclohexanedicarboxylate) (PHCE).

MT-DSC and TOPEM (0.02–5 K min^−1^) cannot give an insight of what happens during quenching, and DSC (5–50 K min^−1^) is limited to slow-crystallizing polymers. FSC is sometimes necessary to emulate the cooling rates experienced by a material during manufacturing, even in the most extreme industrial processing conditions. Since polyesters can crystallize both during the cooling ramp from the melt and during the subsequent heating ramp (cold crystallization), three criteria should be considered: (1) the absence of exothermic events recorded during the cooling ramp (crystallization), (2) the absence of endothermic events recorded during the heating ramp (melting), and (3) the balance of exothermic and endothermic events recorded during the heating ramp (cold crystallization followed by melting).

The results show that, among the four PCHs investigated in this work, the even-numbered ones (PBCE and PHCE) require faster cooling rates compared to the odd-numbered ones (PPCE and PPeCE). Irrespective of the odd or even number of methylene groups introduced in the main structure of the repeating unit, the critical cooling rate decreases as $n_{CH_{2}}$ increases, suggesting that the methylene groups act as defects during crystal formation. This behaviour seems to be specific to alicyclic polyesters, since the opposite is observed in their aromatic counterparts (poly (butylene terephthalate) (PBT) has a critical cooling rate four-decade smaller compared to poly (ethylene terephthalate) (PET)). PCHs with an even number of methylene groups would be the best choice to minimize the cycle time for injection molding without renouncing to developing a semi-crystalline microstructure, because crystallization starts at high temperatures; however, controlling crystal perfection could be challenging, because their crystallization proceeds very fast and the risk of crystalline reorganization upon further heating is high. For vitrification purposes, PCHs with an odd number of methylene groups are recommended, in particular PPeCE, which is the easiest to melt-quench to its fully amorphous state.

## Figures and Tables

**Table 1:** List of poly(alkylene *trans*-1,4-cyclohexanedicarboxylate)s (PCHs) investigated in this study, along with their repeating unit, the molar mass of their repeating unit ($M_{0}$), the number-average molecular weight (${\bar{M}}_{n}$), the weight-average molecular weight (${\bar{M}}_{w}$), the dispersity (*Đ*), the degree of polymerization (DP_*n*_) and the percentage of *cis*-isomers in 1,4-cyclohexanedicarboxylic acid.

Sample	Repeating Unit	$M_{0}$ (g mol^−1^)	${\bar{M}}_{n}$ (g mol^−1^)	${\bar{M}}_{w}$ (g mol^−1^)	*Đ*	DP_*n*_	*cis*-Isomers (%)
PPCE	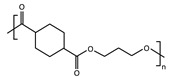	212	62,462	96,657	1.5	295	6.6
PBCE	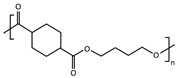	226	68,703	93,382	1.4	304	5.5
PPeCE	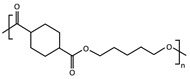	240	57,855	83,592	1.4	241	5.4
PHCE	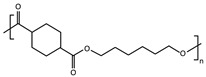	254	38,666	58,734	1.5	152	9.4

PPCE = poly(propylene *trans*-1,4-cyclohexanedicarboxylate); PBCE = poly(butylene *trans*-1,4-cyclohexanedicarboxylate); PPeCE = poly(pentamethylene *trans*-1,4-cyclohexanedicarboxylate); PHCE = poly(hexamethylene *trans*-1,4-cyclohexanedicarboxylate).

**Table 2:** Thermal characteristics of the investigated PCHs extracted from the thermograms in Figure 3. $T_{c}$ is the crystallization temperature measured at the maximum of the exothermic peak observed upon cooling, $\Delta T_{c}$ is the temperature range over which crystallization occurs, $\Delta h_{c}$ is the enthalpy of crystallization, $T_{g}$ is the mid-point glass transition recorded upon heating (insets in Figure 3), $\Delta T_{m}$ is the melting temperature range, $\Delta h_{m}$ is the enthalpy of melting calculated as the algebraic area under the curve in the melting temperature range.

SAMPLE	$T_{c}$	$\Delta T_{c}$	$\Delta h_{c}$	$T_{g}$	$\Delta T_{m}$	$\Delta h_{m}$
	(°C)	(°C)	(J g^−1^)	(°C)	(°C)	(J g^−1^)
PPCE	92.2 ± 0.5	85–106	33 ± 1	8 ± 2	136–160	32 ± 2
PBCE	146.5 ± 0.5	144–150	39 ± 2	7 ± 2	92–171	55 ± 5
PPeCE	25 ± 5	6–51	0.5 ± 0.1	−15 ± 1	60–76	0.6 ± 0.1
PHCE	101.3 ± 0.5	98–105	43 ± 3	−21 ± 2	50–129	57 ± 5

PPCE = poly(propylene *trans*-1,4-cyclohexanedicarboxylate). PBCE = poly(butylene *trans*-1,4-cyclohexanedicarboxylate). PPeCE = poly(pentamethylene *trans*-1,4-cyclohexanedicarboxylate). PHCE = poly(hexamethylene *trans*-1,4-cyclohexanedicarboxylate).

## Data Availability

Dataset available on request from the authors.

## Supplementary Materials

The following supporting information can be downloaded at: https://www.mdpi.com/article/10.3390/polym16192792/s1, Figure S1: H1-NMR spectrum of poly(propylene *trans*-1,4-cyclohexanedicarboxylate) (PPCE). Figure S2: H1-NMR spectrum of poly(butylene *trans*-1,4-cyclohexanedicarboxylate) (PBCE). Figure S3: H1-NMR spectrum of poly(pentamethylene *trans*-1,4-cyclohexanedicarboxylate) (PPeCE). Figure S4: H1-NMR spectrum of poly(hexamethylene *trans*-1,4-cyclohexanedicarboxylate) (PHCE).
