# Supplementary material for: Critical Cooling Rate of Fast-Crystallizing Polyesters: The Example of Poly(alkylene trans-1,4-cyclohexanedicarboxylate)
Source: Polymers (Basel). 2024 Oct 1;16(19):2792. doi: 10.3390/polym16192792 (PMC11478611; doi:10.3390/polym16192792)
Supplement: Supplementary file 1 [file polymers-16-02792-s001.zip › polymers-3224133-supplementary.pdf]

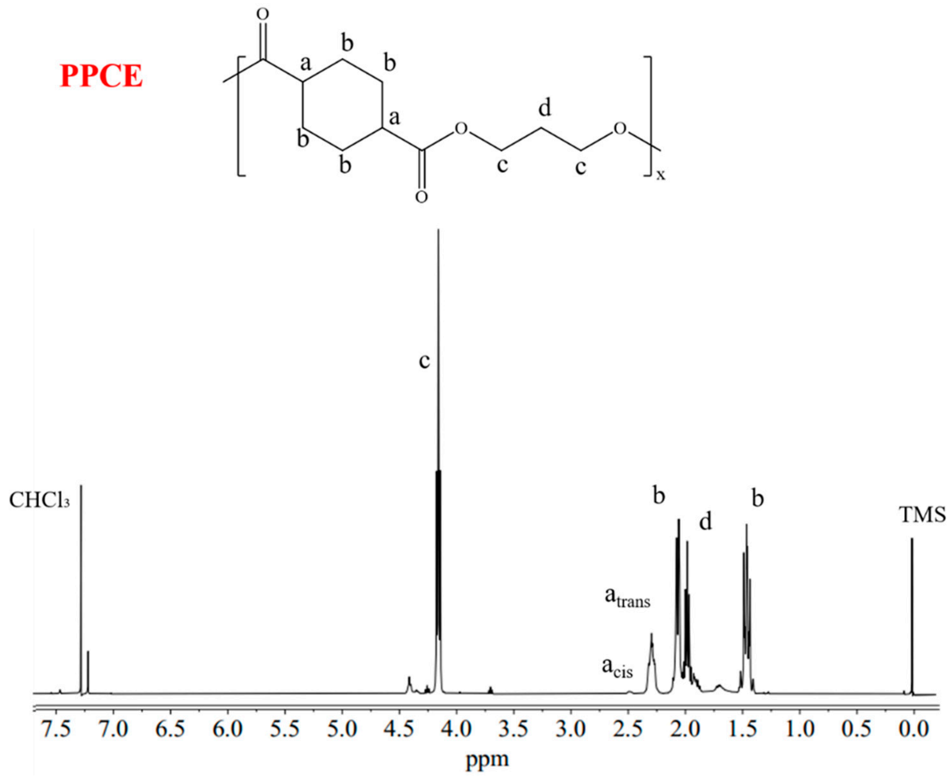

Figure S1. ^{1}H-NMR spectrum of poly(propylene *trans*-1,4-cyclohexanedicarboxylate) (PPCE).

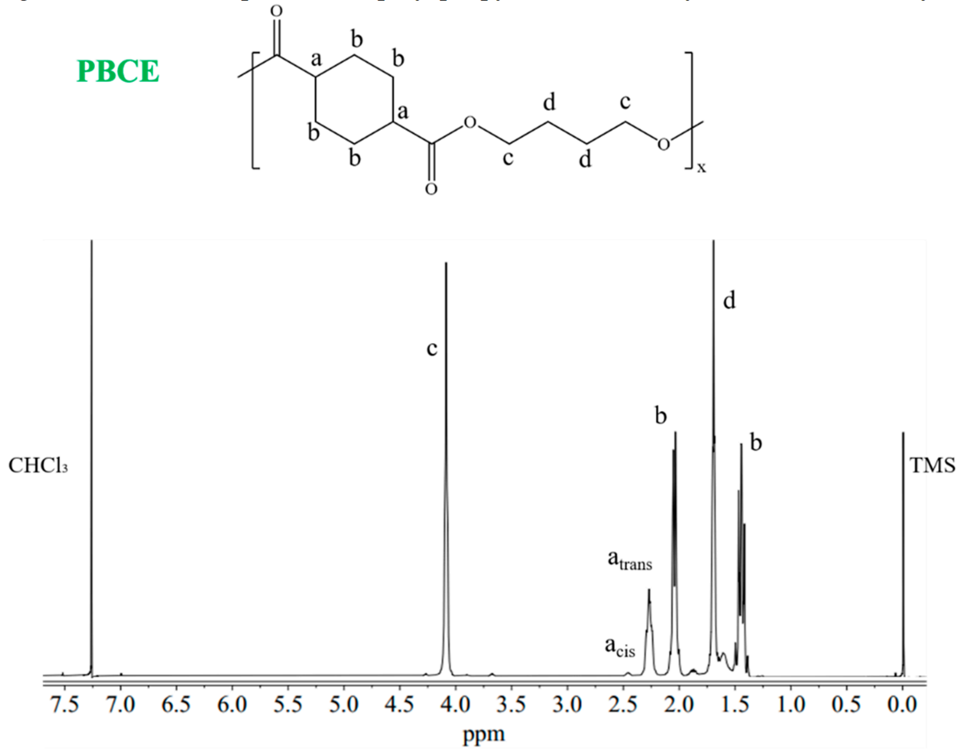

Figure S2. ^{1}H-NMR spectrum of poly(butylene *trans*-1,4-cyclohexanedicarboxylate) (PBCE)

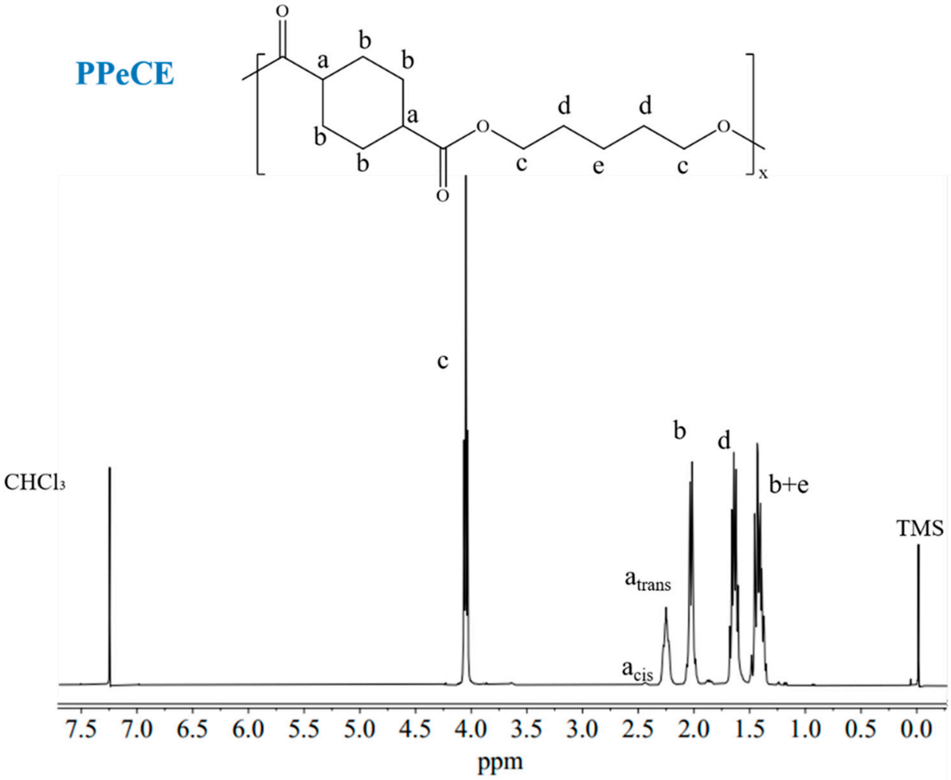

Figure S3. ^{1}H-NMR spectrum of poly(pentamethylene *trans*-1,4-cyclohexanedicarboxylate) (PPeCE)

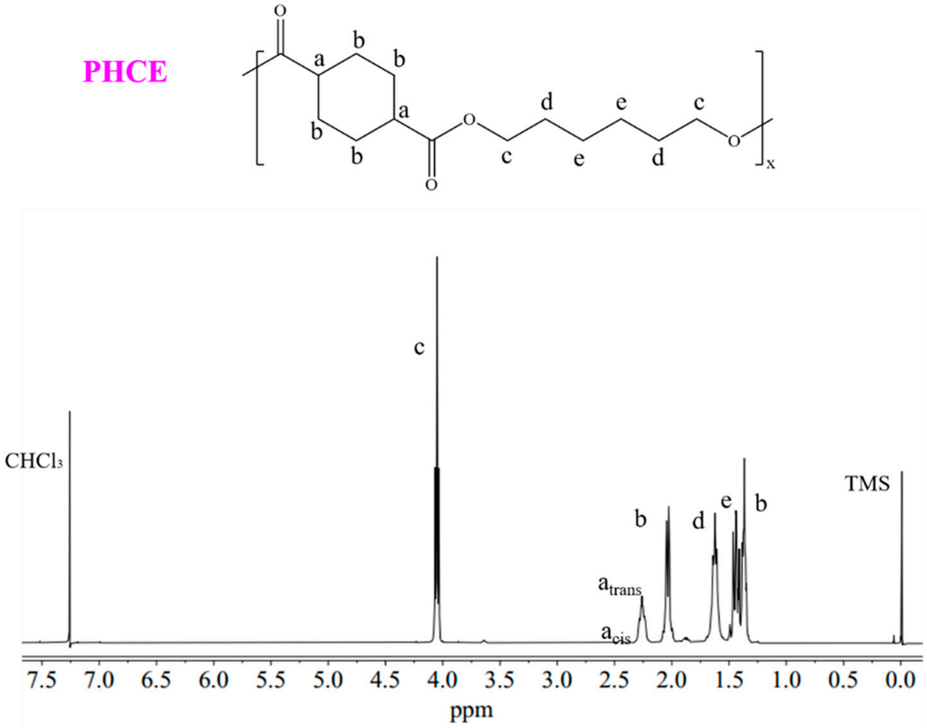

Figure S4. ^{1}H-NMR spectrum of poly(hexamethylene *trans*-1,4-cyclohexanedicarboxylate) (PHCE).
